# Three novel hub genes and their clinical significance in clear cell renal cell carcinoma

**DOI:** 10.7150/jca.35223

**Published:** 2019-11-01

**Authors:** He Xiao, Ping Chen, Guang Zeng, Deqiang Xu, Xinghuan Wang, Xinhua Zhang

**Affiliations:** 1 Urological Surgery, Wuhan Children's Hospital (Wuhan Maternal and Child Healthcare Hospital), Tongji Medical College, Huazhong University of Science & Technology, Wuhan 430017, China; 2 Department of Urology, Zhongnan Hospital of Wuhan University, Wuhan 430071, P.R. China; 3 Biomedical Engineering, Stony Brook University, New York 11790

**Keywords:** weighted gene co-expression network analysis (WGCNA), clear cell renal cell carcinoma (ccRCC), survival, prognosis, biomarkers

## Abstract

***Purpose.*** To investigate the association of biomarkers correlated with clinical stages and survival of clear cell renal cell carcinoma (ccRCC).

***Methods.*** The GSE36895 dataset was downloaded and differentially expressed or methylated genes were analyzed. Hub genes were identified with weighted gene co-expression network analysis (WGCNA) and protein-protein interaction network (PPI), and validated with TCGA database and our own tissues. The biological processes of hub genes were further explored by functional enrichment analysis. Survival analyses were also performed. The underlying mechanisms for ccRCC development were detected with Gene set enrichment analyses.

***Results.*** A total of 1624 differentially expressed genes were analyzed by WGCNA and 6 co-expressed gene modules were identified. Three hub genes (EHHADH, ACADM and AGXT2) were met the criterion of both WGCNA and PPI networks analysis, which showed highest negative association with pathological T stage (r = - 0.45, p = 0.01) and tumor grade (r = - 0.45, p = 0.01). The downregulation of these hub genes was validated with using both TCGA database and samples harvested at our institute The biological processes that hub genes involved, such as metabolic process (p = 9.63E - 09), oxidation-reduction process (p = 1.05E - 08) and oxidoreductase activity (p = 1.72E - 04), were revealed. Survival analysis showed a higher expression or lower methylation of these hub genes, a longer survival of ccRCC patients. ccRCC samples with higher expression of hub genes were enriched in gene sets correlated with signaling like biosynthesis of unsaturated fatty acids, butanoate metabolism, and PPAR signaling pathway.

***Conclusions.*** We identified three novel tumor suppressors associated with pathological T stage and overall survival of ccRCC. They might be potential as individualized therapeutic targets and diagnostic biomarkers for ccRCC.

## Introduction

Kidney cancer is a common malignant illness 1. Around 90% pathological type of renal cancers is renal cell carcinoma (RCC), majority of which are subtyped as clear cell renal cell carcinoma (ccRCC) 2. Based on the size and metastasis of tumor, the degree of invasion external of the kidney and the involvement of lymph node, ccRCC is classified into pathological T stages, pathological N stage, metastasis and clinical stage 3. It is well known that the prognosis of this disease is correlated to the pathological stage and the five-year survival rates of these four pathological stages are 95%, 88%, 59% and 20% for the aforementioned stages, respectively 4. Indeed, localized ccRCC can be cured with radical nephrectomy but the prognosis is poor when the disease turned to be metastatic. In case of the late staged ccRCC, traditional chemotherapies are usually tolerant. In recent decades, different oncogenes related to ccRCC had been identified with high-throughput microarray technology 5-7. Treatments targeted on these discovered genes have been proved more effective than chemotherapies, duo to the target specificity and low adverse effect 8. A number of targeted therapies have been accepted for clinical use, such as anti-vascular endothelial growth factor (VEGF) antibodies, mammalian target of rapamycin (mTOR) and multi-kinase inhibitors 9. Although patients' survival have been ameliorated with these new treatments, median overall survival and progression-free are virtually 2 years and most cases finally become surrender and resistance 8. Ignorance of interconnection between genes could contribute to the failure of these targeted therapies, as carcinogenesis progression is not only the consequence of deregulation of some tumor suppressors or oncogenes but also the result of complex molecular mechanisms, including the strong interconnection between genes with similar expression patterns. Therefore, to achieve effective individualized treatments for ccRCC, more therapeutic targets should be identified and their interconnection should be determined.

Langfelder et al. firstly used weighted gene co-expression network analysis (WGCNA) to explore a thorough association between different gene sets or between gene sets and clinical characteristics 10. With emerging plenty of microarray or RNA sequencing data, WGCNA has been widely performed to filter modules and hub genes that are correlated to clinical features like grade, metastasis and tumor stages among various tumor types such as hepatocellular carcinoma 11 and papillary renal cell carcinoma 12. With regarding to ccRCC, our center analyzed a microarray data with WGCNA and discovered six hub genes (*CCNB2*,* CDC20*,* CEP55*,* TOP2A*,* KIF20A* and* UBE2C*) that were highly correlated with pathologic stage of ccRCC 13. Also in our center, Chen et al. identified a hub gene *FCER1G* through co-expression network analysis of another microarray data and demonstrated this hub gene had connection with progression and prognosis of ccRCC via influencing immune-related pathways 14. In current study, we downloaded a different microarray dataset and tried to build a co-expression network with a systematical biology process of WGCNA. Furthermore, ccRCC and adjacent normal kidney tissues wer harvested to verify the bioinformatic analysis. We aimed to seek and validate other different hub genes which are associated with clinical stages and survival of ccRCC 15-17.

## Materials and Methods

### Data collection

GSE36895 microarray dataset, containing 29 homo ccRCC tissues and 23 homo normal kidney tissues, was downloaded from Gene Expression Omnibus (GEO) database (http://www.ncbi.nlm.nih.gov/geo/) for constructing co-expression networks and exploring hub genes. Patient's clinical information of ccRCC tissues included age, gender, different grades (I -- Ⅳ), pathological T stages (I -- Ⅳ), pathological N stages (I -- Ⅲ), metastasis (M0 and M1) and clinical stages (I -- Ⅳ). We also downloaded RNA-sequencing dataset with detailed clinical information from The Cancer Genome Atlas (TCGA) database (https://genome-cancer.ucsc.edu/) to validate the gene expression based on the RNA-sequencing technology of IlluminaHiseq.

### Data preconditioning

The raw data were background corrected, log2 transformed and quantile normalized by Robust Multi-array Averaging (RMA). The "Affy" R package was used to summarize median polish probesets which were annotated with the files of Affymetrix annotation. Finally, sample clustering was applied to evaluate the quality of GSE36895 dataset.

### Differential expression genes (DEGs) screening

DEGs between ccRCC and normal renal tissues were screened using R software based on "limma" R package at a preset threshold with |log2 fold change (FC)| > 1 and p value < 0.05.

### Co-expression network construction

After verifying the qualification of DEGs' expression data, a co-expression network was set for the DEGs using R software based on the "WGCNA" R package. Pearson's correlation matrices were conducted and a weighted adjacency matrix were performed by a formula amn = |cmn|^β^ (cmn represents Pearson's correlation between genes, amn represents adjacency between genes and the soft-thresholding parameter (β) was able to magnify the correlation between genes through enhancing high correlations and weakening low correlations). In current study, β = 6 was chosen to guarantee a scale-free network. Subsequently, the adjacency was transformed into topological overlap matrix (TOM) and identified modules including similar genes by hierarchically clustering genes 18. To categorize genes with analogous expression into gene modules, an average linkage hierarchical clustering was carried out based on TOM dissimilarity measure with a minimal gene size of 30 for constructing a dendrogram 19. Finally, a cut-line was selected for module dendrogram and merged some modules after dissimilarity of estimated module eigengenes being evaluated.

### Discovering the interesting module

Module eigengenes (MEs) were considered as the most principal component and all genes were summarized into a single characteristic expression profile. The interesting module was identified by calculating the relevance between MEs and clinical feature. The log10 transformation of the p value was defined as gene significance (GS) and the average GS for all genes in the module was defined as the module significance (MS). The module with the highest MS score was chosen as the one related to clinical feature.

In order to investigate the possible mechanism of the association between the interesting module genes and correlated clinical characters, all genes in brown module were uploaded into the DAVID database and analyzed by GO functional enrichment analysis with a cutoff criterion of false discovery rate (FDR) < 0.01.

### Identification and validation of hub genes

For interesting module, the hub genes were defined based on module connectivity (Pearson's correlation of module membership > 0.8) and clinical characteristic relation (Pearson's correlation of GS > 0.2). Moreover, protein-protein interaction (PPI) network was built through putting all relevant genes from the module into the Search Tool for Interacting Genes' Retrieval (STRING). The common hub genes in both co-expression network and PPI network were regarded as “real” hub genes for further analyses.

### Efficacy evaluation and survival analysis

TCGA data were utilized to evaluate the association between the expression of the most interesting hub genes and the different pathological stages of ccRCC using Gene Expression Profiling Interactive Analysis (GEPIA) database (http://www.gepia.cancer-pku.cn). The survival rate analysis was conducted based on the TCGA database for the assessment of the identified genes' effects on the prognosis of ccRCC patients. Firstly, patients with mRNA data were classified in two different categories in accordance with each gene's median expressions (low vs. high). Patients with methylation data were similarly analyzed. Secondly, analysis was conducted on patients with both mRNA expression and different ccRCC grades data. Finally, we performed Kaplan-Meier survival analysis and the log-rank test by adopting the “survival” R package. One-way analysis of variance (ANOVA) and paired 2-tailed Student's t tests were used to analyze the statistical significance of differences of data.

### Gene set enrichment analysis (GSEA)

Two categories (high vs. low) of the most interesting hub genes in 539 ccRCC patients were classified and the median value of gene expression was applied as the cut-off point. GSEA (http://software.broadinstitute.org/gsea/index.jsp) was carried out to investigate potential functions of the most interesting hub genes with a cut-off criteria of |Enrichment score (ES) | > 0.5 and p value < 0.05.

### Human ccRCC and adjacent normal kidney tissues

ccRCC and adjacent normal kidney tissues (n = 15) were obtained from patients undergoing laparoscopic nephrectomy at Zhongnan Hospital of Wuhan University. Two pathologists independently confirmed the histological diagnosis. Half of each specimen was immediately fixed in 4% PFA (paraformaldehyde) and half stored in liquid nitrogen. The use of these ccRCC specimens was approved by the Ethics Committee at Zhongnan Hospital of Wuhan University, and informed consent was obtained from all patients.

### Total RNA extraction and real-time RT-PCR

Total RNA was isolated from the frozen tissues using Takara RNAiso Plus (Takara Bio. Inc., Otsu, Shiga, Japan) according to the manufacturer's protocol. Genomic DNA (gDNA) was removed and cDNA was reverse-transcribed using Takara PrimeScriptTM RT reagent Kit with gDNA Eraser (Takara Bio. Inc., Otsu, Shiga, Japan) in a T100TM Thermal Cycler System (BioRad, USA). The experimental protocol utilized was first gDNA removal (42 °C, 2 min), followed by reverse transcription (37 °C 15 min, 85 °C 5 s). Subsequently, all samples were amplified by a 25 μl reaction volume in a CFX96TM Real-time PCR Detection System (BioRad, USA), using SYBR® Premix Ex TaqTM Ⅱ (Takara Bio. Inc., Otsu, Shiga, Japan). All samples were run in triplicate. The seven identified genes were investigated. The amplification program was repeated for 40 cycles. For relative quantification, gene expression was normalized to expression of GAPDH housekeeping gene and compared by 2^-ΔΔCT^ method.

### Immunohistochemistry

For immunohistochemistry, sections were deparaffinized in xylene, followed by graded alcohols. Antigen retrieval was performed in 10 mM sodium citrate buffer (pH 6.0) and heated to boil. Sections were kept in boiled buffer for 2 min. Endogenous peroxidase activity was blocked by using 3% H2O2 solution at room temperature for 10 min. Then sections were incubated with 15% normal goat serum for 15 min at room temperature to block non-specific binding. Primary antibody was applied to the sections on the slides and incubated in a humidified chamber at 4 ◦C overnight. Then the sections were stained by routine immunohistochemistry methods.

## Results

### Different expression of genes screened

**Figure 1:**
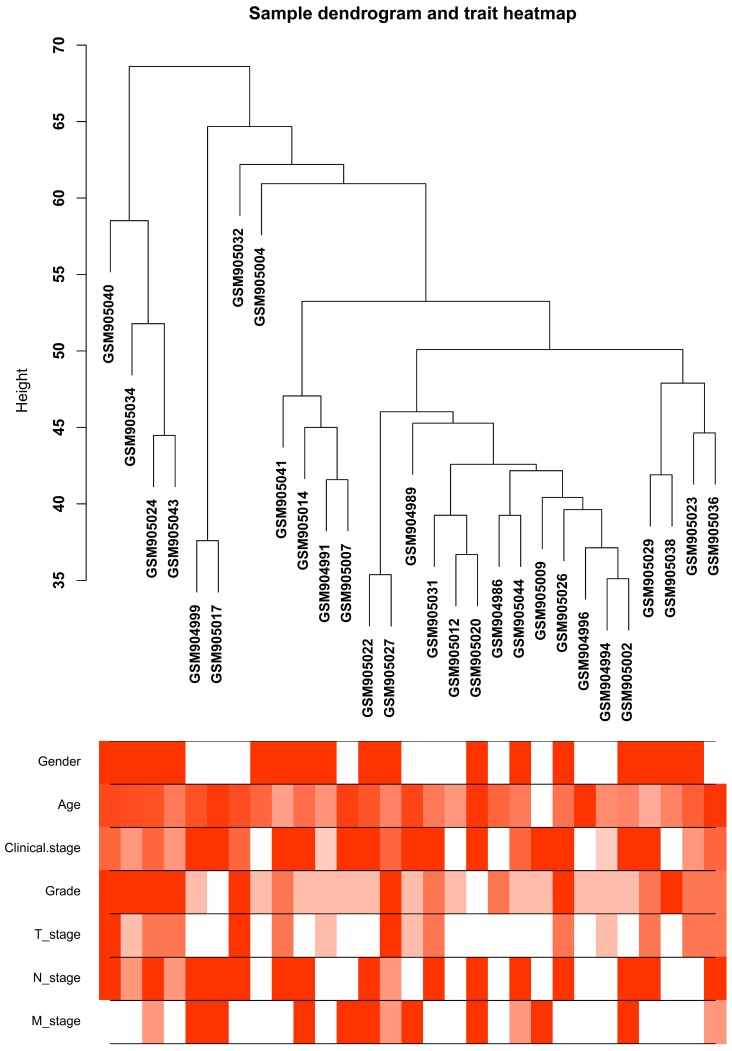
**Clustering dendrogram of the clinical traits and 29 tumor samples.** The clustering is based on differential expression genes (DEGs) data in ccRCC tumor samples compared to non-tumor samples. The red color represents female and metastasis and the intensity of the color is proportional to higher tumor grade and pathological stage as well as older age.

After quality evaluation and data preprocessing, the expression matrix was acquired from the 52 samples of GSE36895 dataset (Figure 1). With the |log2FC| > 1and p value < 0.05, a sum of 1624 DEGs (886 down-regulated and 738 up-regulated) were selected for subsequent analyses.

### Weighted co-expression network construction and key modules identification

**Figure 2:**
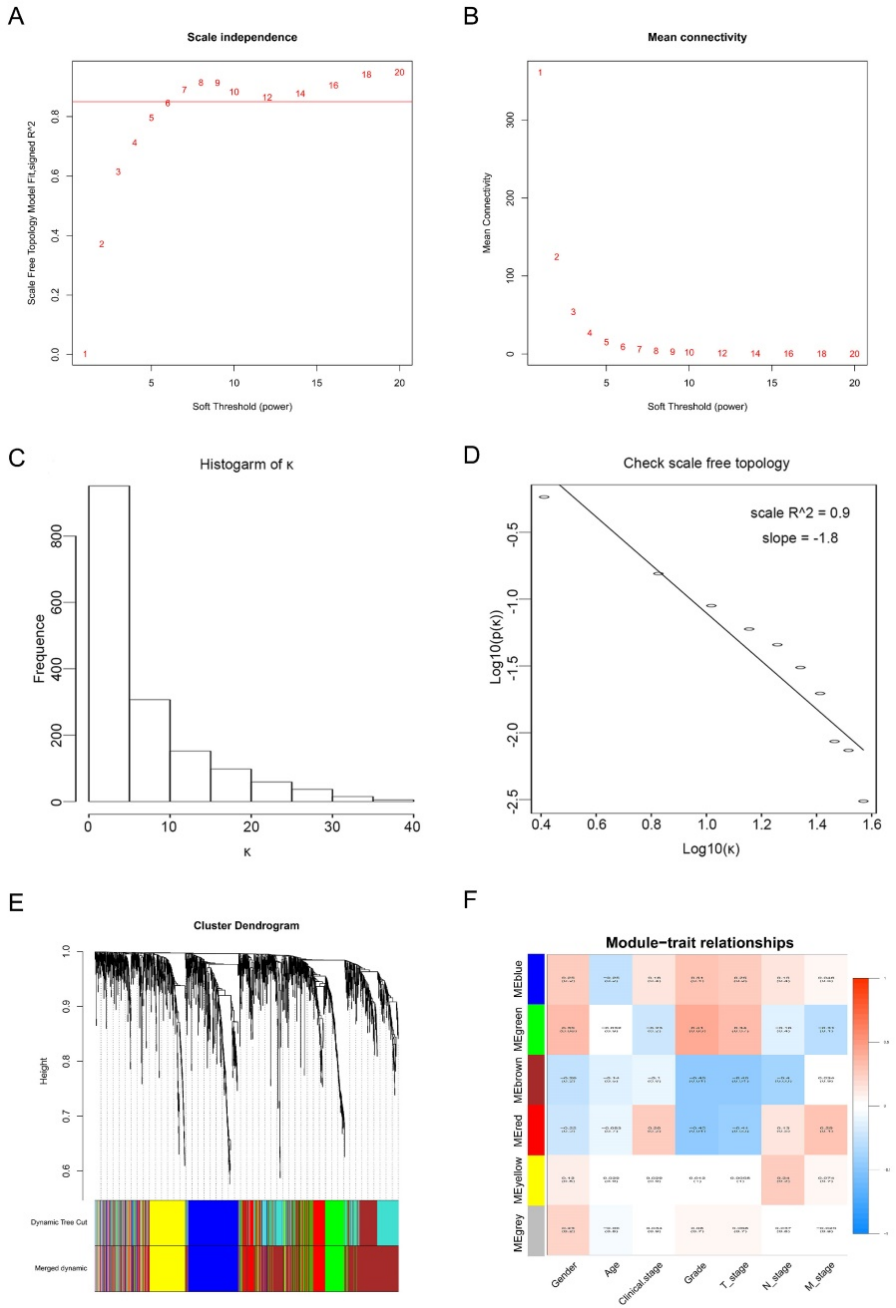
** Calculation of soft-thresholding power value in the weighted gene co-expression network analysis (WGCNA).** (**A**) Analysis of the scale-free fit index for various soft-thresholding powers (β). (**B**) Analysis of the mean connectivity for various soft-thresholding powers. (**C**) Histogram of connectivity distribution when β = 6. (**D**) Checking the scale-free topology when β = 6.** (E)** Clustering dendrogram of the DEGs clustered. **(F)** Heatmap of the correlation between module eigengenes and clinical traits of ccRCC.

Twenty-nine ccRCC samples with clinical information were included for the co-expression analysis with β = 6 used as the soft-thresholding to guarantee a free scale network (scale free R^2^ = 0.85) (Figures 2A - D). A sum of 6 different modules were identified (Figure 2E). The highest absolute MS score in the Module-feature relationship was found between pathological T stage and brown module (r = - 0.45, p = 0.01; Figure 2F), which was chosen for the subsequent analyses. Interestingly, the brown module was also found to be associated with pathological lymph nodes stage (r = - 0.40, p = 0.03) and tumor grade (r = - 0.45, p = 0.01).

**Figure 3:**
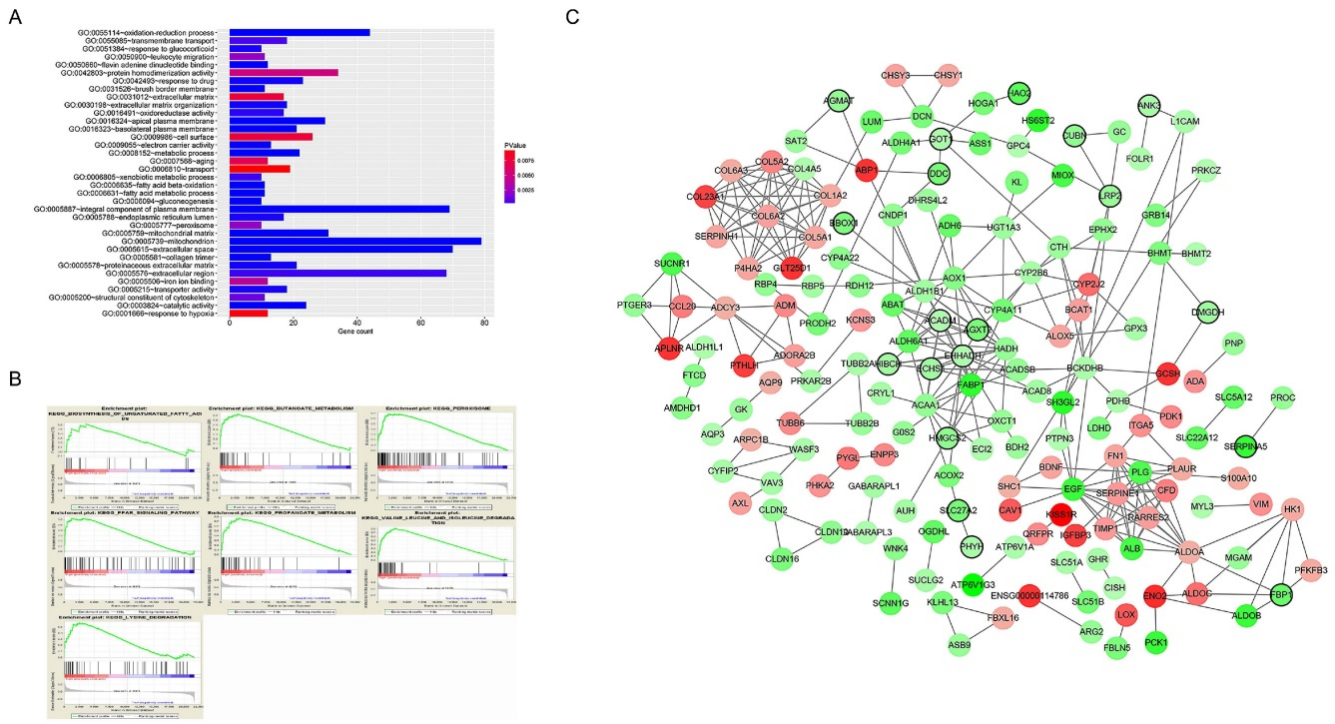
** Functional enrichment and protein-protein interaction (PPI) network.** The x-axis displays the amount of gene and the y-axis displays the GO terms. The -log10 (p value) of each term is colored following the legend. The intensity of the color in each node is proportional to gene expression compared to non-tumor samples (down-regulation in green and up-regulation in red). Network hub genes identified by WGCNA are represented by the nodes with bold circle.

A total of 565 genes associated biological relevance in brown module were investigated utilizing DAVID database for GO (Gene Ontology) analyses. As shown in Figure 3A, 36 enriched biological procedures were found to be possible mechanisms of how the brown module genes impact on pathological T stage, such as metabolic process (p = 9.63E - 09), oxidation-reduction process (p = 1.05E - 08), oxidoreductase activity (p = 1.72E - 04) and fatty acid beta-oxidation (p = 1.45E - 06).

### GSEA of hub genes

To explore the underlying roles of these three hub genes, we conducted GSEA to map into KEGG (Kyoto Encyclopedia of Genomes and Genes) pathways database. According to the cut-off criteria with |ES| > 0.5 and p value < 0.05, a sum of 20 significant gene sets were found and majority of which focused on metabolic relevant pathways. Six representative pathways were “Biosynthesis of unsaturated fatty acids”, “Butanoate metabolism”, “Peroxisome”, “PPAR signaling pathway”, “Propanoate metabolism” and “Valine leucine and isoleucine degradation” (Figure 3B).

### Identification of hub gene

In current study, 18 high connective genes in brown module were selected as hub genes (Table 1). Moreover, we conducted a PPI network for all genes in brown module using Cytoscape software and the genes associated with more than 7 nodes were regarded as hub node genes (Figure 3C). The three common hub genes (*EHHADH*, *ACADM* and *AGXT2*), met both criterions in co-expression and PPI networks, were selected as "true" hub genes for further validation (Table 1).

### Hub gene validation

**Figure 4:**
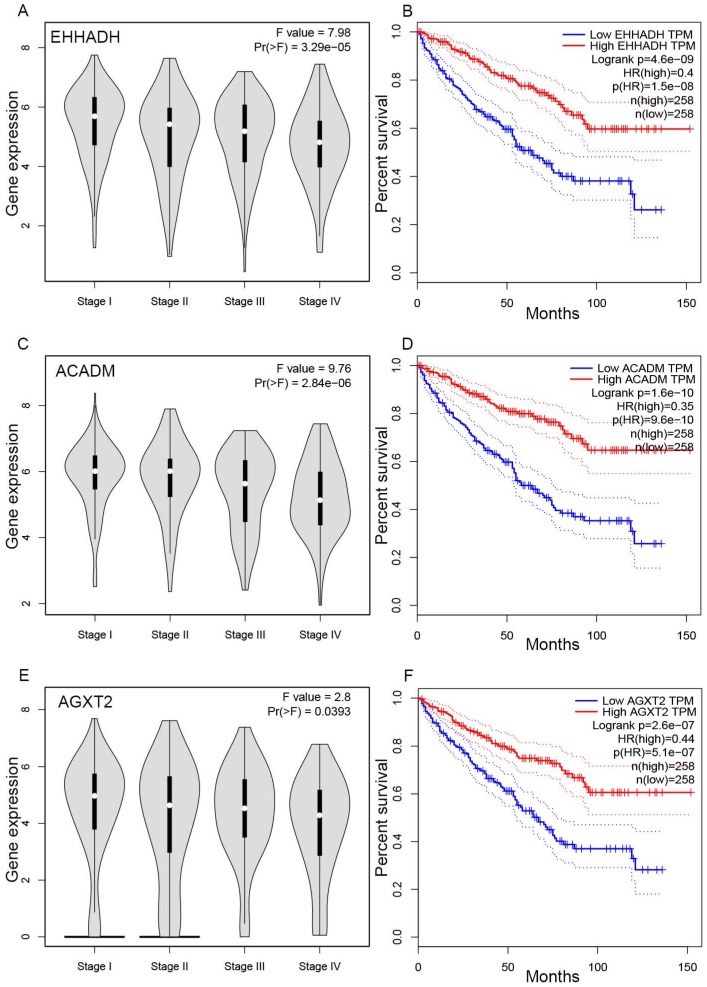
** Validation of hub genes.** Panels** A**,** C** and** E** show the correlation of *EHHADH*, *ACADM* and *AGXT2* expression with the pathological stage of ccRCC, respectively (based on microarray data of TCGA). Panels** B**,** D** and** E** show survival analyses of *EHHADH*, *ACADM* and *AGXT2* genes in the TCGA data set, respectively.

**Figure 5:**
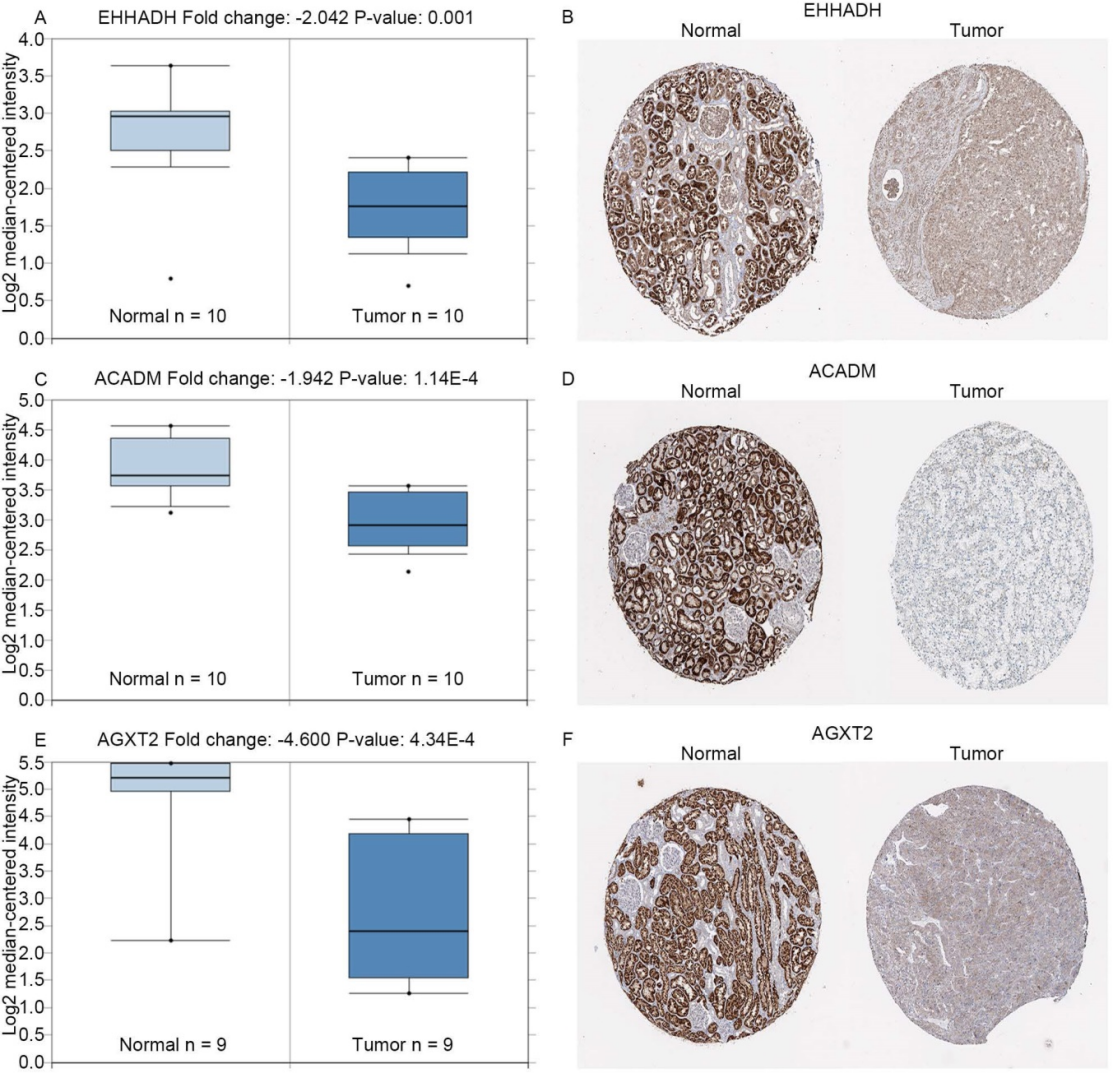
** Expression of hub genes.** Panels **A**, **C** and **E** show the mRNA expression of *EHHADH*, *ACADM* and *AGXT2* in ccRCC tissues compared to normal kidney tissues based on Oncomine database, respectively. Panels **B**, **D** and **F** show the protein expression of *EHHADH*, *ACADM* and *AGXT2* in ccRcc tissues compared to normal kidney tissues based on The Human Protein Atlas database, respectively.

**Figure 6:**
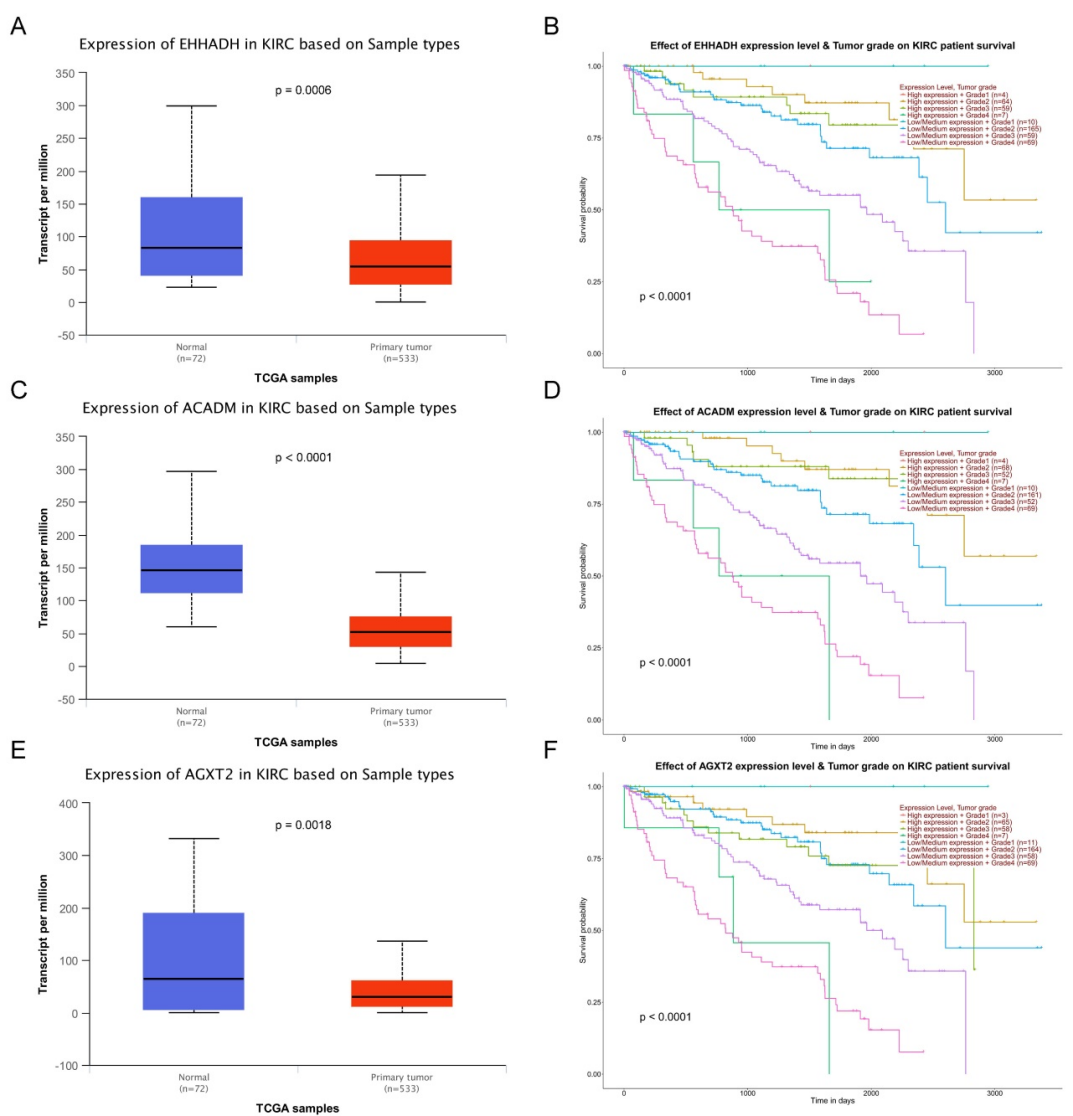
** Validation of expression and survival analysis of three hub genes expression combined with tumor grades in TCGA database.** Panels A, C and E show the mRNA expression of *EHHADH*, *ACADM* and *AGXT2* in ccRCC tissues compared to normal kidney tissues based on TCGA. Panels B, D and F show survival analyses of *EHHADH*, *ACADM* and *AGXT2* genes expression combined with tumor grades in the TCGA data set, respectively.

The complete data of 539 ccRCC patients in the TCGA dataset were carried out to validate hub genes, demonstrating that all three hub genes exhibited a substantial negative correlation with different ccRCC stages, consistent with above analyses of GSE36895 microarray dataset (Figures 4A, C, E). In addition, based on TCGA data, significantly longer overall survival times were shown in patients with higher expression of these three hub genes, indicating that *EHHADH*, *ACADM* and *AGXT2* were prognostic biomarkers for ccRCC (Figures 4B, D, F). Furthermore, as shown in Figure 5, both protein and mRNA expression of these hub genes were significantly lower in ccRCC tissues compared to normal ones, which were provided and confirmed by The Human Protein Atlas and Oncomine databases. To assess the roles of these three hub genes in ccRCC, gene expression validations were performed, and all of three hub genes were also downregulated in the TCGA database (Figure 6A, C, E) and longer overall survival duration was also found in cases of lower expression at each tumor grade. (Figure 6B, D, F).

### Association of three hub genes methylation level with the prognosis of ccRCC

**Figure 7:**
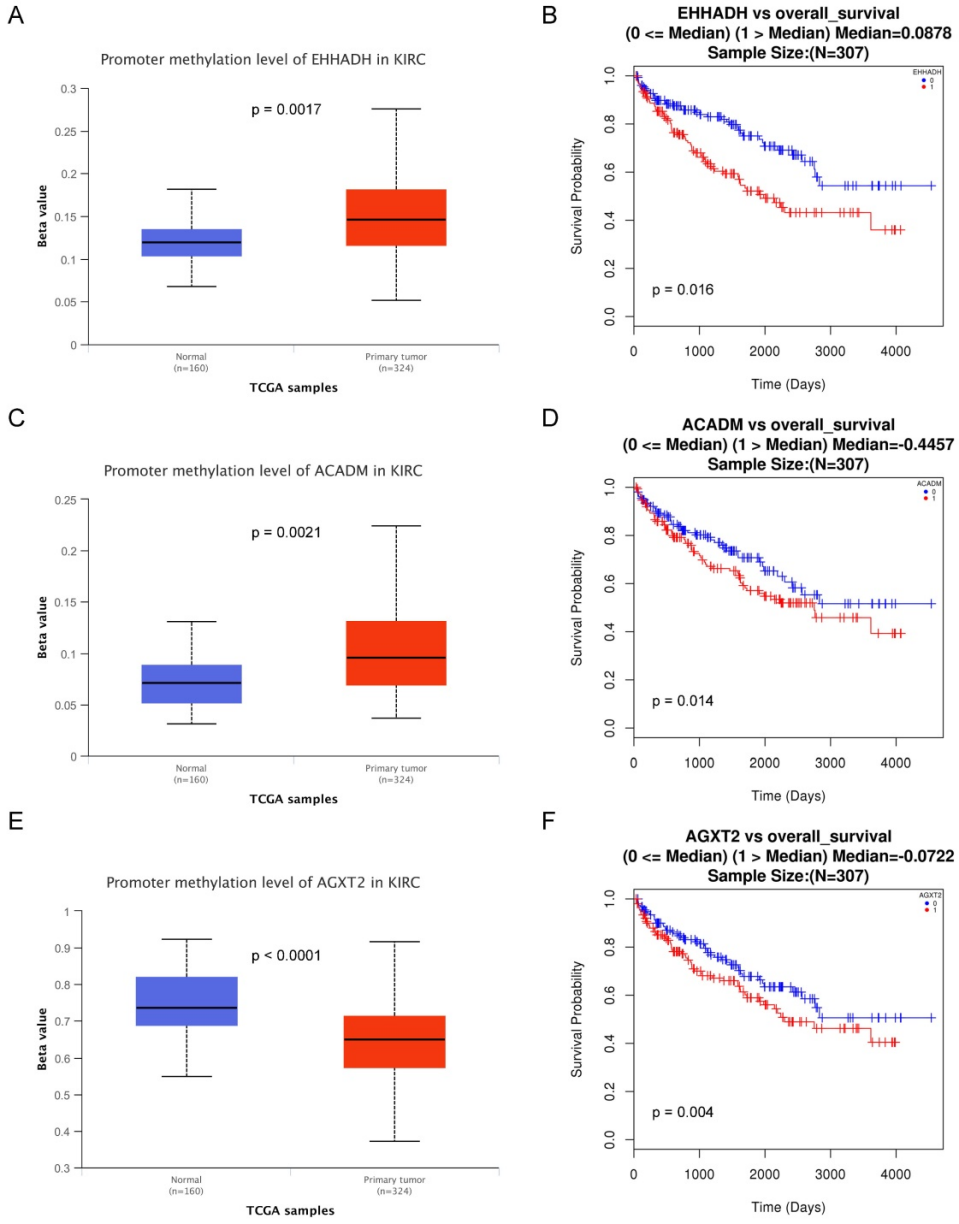
** Methylation status and survival analysis of three hub genes.** Box plot showing the methylation levels of the three genes, using data from the TCGA database. The x-axis shows the number of the normal samples and ccRCC samples. The y-axis shows beta value of gene methylation which were shown in Figure 7A, C and E. The Kaplan-Meier survival curve was plotted. It revealed that the overall rates of survival for the patients with 2 hyper-methylated genes (*EHHADH* and *ACADM*) and 1 hypo-methylated gene *AGXT2* were significantly lower, which were shown in Figure 7B, D and F.

The methylations of the three hub genes identified above were further analyzed with TCGA database. As shown in Figure 7(A, C and E), *EHHADH* and *ACADM*) were found hyper-methylated while *AGXT2* hypo-methylated in tumor tissues. The survival curves were drawn to evaluate the association between three hub genes methylation levels and the prognosis of ccRCC, respectively. The two hyper-methylated genes (*EHHADH* and *ACADM*) were associated a shorter OS duration, but the hypomethylated one (*AGXT2*) also showed a shorter OS (Figure 7B, D, F).

### Expression of the identified hub gene in ccRCC and normal tissues obtained in our institute

**Figure 8:**
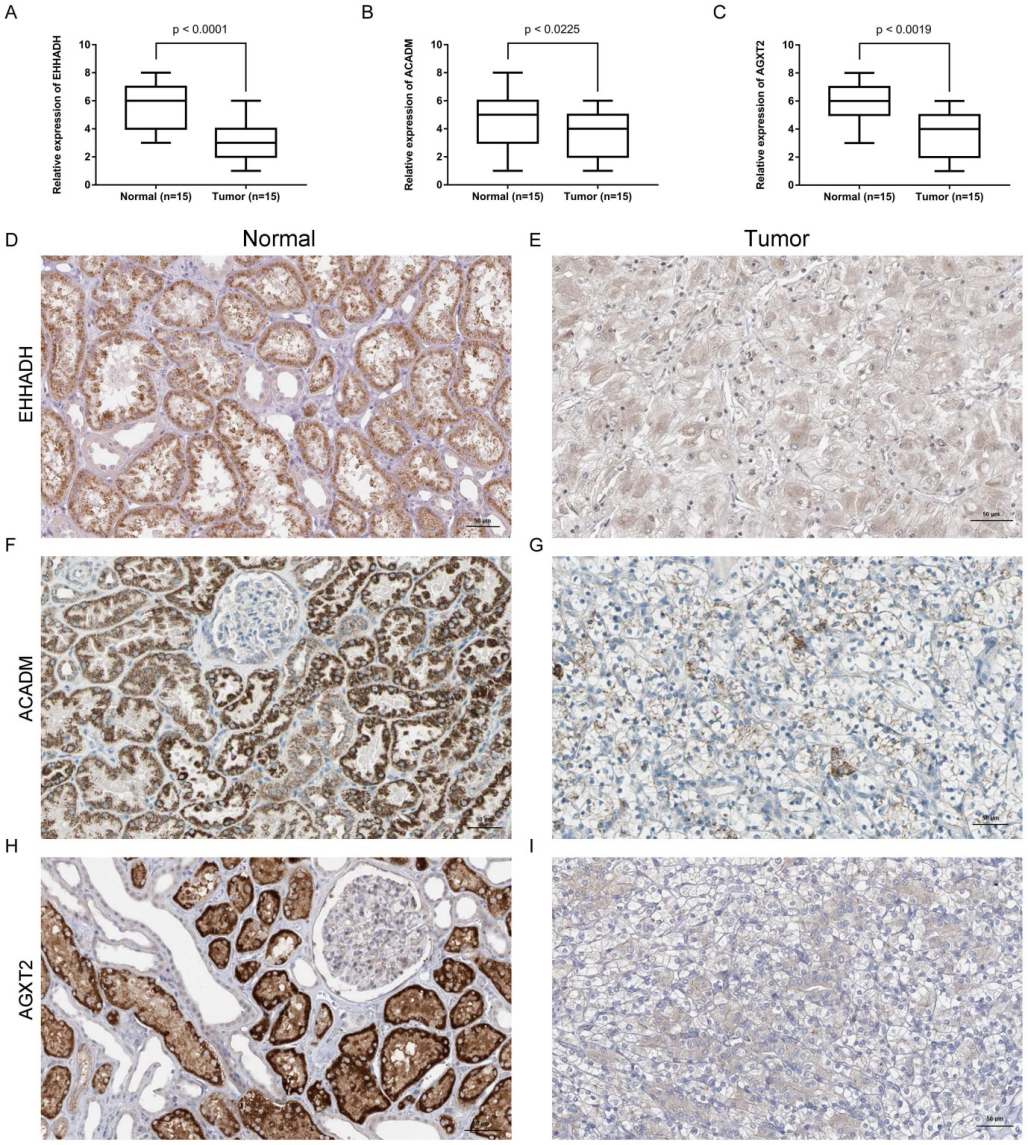
** Expression and localization of three genes in normal kidney tissues and ccRCC tissues.** (A - C). Transcriptional levels of three genes in ccRCC tissues and normal ones. (D and E). Immunohistochemistry of *EHHADH* in in ccRCC tissues and normal ones. The magnification is × 200. (F and G). Immunohistochemistry of *ACADM* in ccRCC tissues and normal ones. The magnification is × 200 (H and I). Immunofluorescence of *AGXT2* in ccRCC tissues and normal ones.

Expression of *EHHADH*,* ACADM* and *AGXT2* mRNA were determined using quantitative real time RT-PCR and immunohistochemistry between ccRCC samples and normal ones. *EHHADH* expression was most significantly downregulated at the transcription level (p < 0.0001) in ccRCC. Real time RT-PCR also showed that for the other two genes that *ACADM* (p = 0.0225) and *AGXT2* (p = 0.0019) the mRNA expression was significantly altered in the ccRCC samples (Figure 8A, B, C). As shown in Figure 8 (D - I), in normal tissue, all three genes were mainly present in in renal tubules and partly present in the glomeruli. In ccRCC, the localization is similarly with that in normal ones, the immune positivity is significantly lower than that of normal. That is to say, immunohistochemistry also confirms that the expression of the three genes *EHHADH*,* ACADM* and* AGXT2* in ccRCC is lower than that in normal tissues.

## Discussion

Current study identified three novel hub genes (*EHHADH*, *ACADM* and *AGXT2*) through analyzing the co-expression and PPI networks. Our data further demonstrated these hub genes showed a negative relationship with different ccRCC stages, which having impact on overall survival. Also, the methylation of these hub genes was found negatively corelated with survival. In addition, it was found these three hub genes might play important roles in ccRCC prognosis through metabolic related pathways.

GSE36895 data were used. There are 29 cases of ccRCC and 23 normal kidney tissues involved 1624 DEGs (886 down-regulated and 738 up-regulated). These genes were further analyzed with WGCNA through constructing a gene co-expression network based on the expression similarity among samples. WGCNA have been used to discover complex disease related genes, biological pathways and neoplasm treatment targets of Alzheimer's disease, osteoporosis and hyperlipidemia. Also, in our center, Yuan et al. analyzed GSE40435 and discovered six hub gene that were highly correlated with pathologic stage of ccRCC 13. Chen et al. identified a hub gene *FCER1G* which might regulate immune-related pathways to tumorigenesis through co-expression network analysis of another microarray data GSE66272 14. In present study, a larger ccRCC sample size was analyzed with WGCNA and 18 hub genes were screened from brown module. Three common hub genes (*EHHADH*, *ACADM* and *AGXT2*), met both analyses of co-expression and PPI networks, were regarded as “real” hub genes and were further validated. Our data indicated the three hub genes had high connection with clinical prognosis as well as vital biological processes.

In the GEPIA database, we found a trend that the expression of *EHHADH*, *ACADM* and *AGXT2* was negatively correlated with the pathological stages of ccRCC (Figures 4A, C, E), which illustrated the critical role of these three hub genses in the progression of ccRCC. In addition, these hub genes were found negatively corelated with survival at tumor grade (Figure 6). Indeed, the Oncomine database found a significant lower expression of *EHHADH*, *ACADM* and *AGXT2* in ccRCC tissues than normal kidney tissues (Figures 5A, C, E). In addition, immunohistochemistry staining in The Human Protein Atlas database showed that the expression of *EHHADH*, *ACADM* and *AGXT2* proteins were also significantly lower in renal carcinoma compared to normal kidney (Figures 5B, D, F). Consistently, the lower expression of the 3 hub genes was determined both at the mRNA and protein levels with using tissues harvested from our institute (Figure 8). Thus, our study indicated a negatively role of these three hub genes in clinical stages of ccRCC development. Moreover, we performed survival analysis to validate if these three hub genes were associated with patient prognosis (Figures 4B, D, F). According to the GEPIA database, we found that a lower expression of *EHHADH*, *ACADM* and *AGXT2*, a shorter overall survival time. Also, the hyper-methylation of these hub genes was found negatively associated with survival (Figure 7). However, the hypo-methylated gene* AGXT2* also showed a shorter OS, which will need further investigation In line with previous report, hyper-methylated and lower expressed genes showed worse prognosis. Therefore, *EHHADH*, *ACADM* and *AGXT2* could be suggested as protective tumor suppressors for ccRCC.

*EHHADH* (3-hydroxyacyl CoA dehydrogenase and enoyl-CoA hydratase) encodes a bifunctional enzyme that is one of the four enzymes of peroxisomal β-oxidation pathway 20. Suto K et al. and Cablé S et al. found that *EHHADH* was lower expressed in hepatocellular carcinoma and colon carcinoma and could be used as a potential prognostic marker 21, 22. *ACADM* (medium-chain acyl-CoA dehydrogenase) could catalyze the first dehydrogenation in fatty acyl-CoA beta-oxidation in mitochondria 23 and *ACADM* insufficiency might impact on the medium-chain fatty acids which exist abundantly in the beta-oxidation pathway that would indirectly influence the triglycerides metabolism 24, 25 and play a significant role in cell apoptosis through the function of light chain. *AGXT2* (alanine-glyoxylate aminotransferase 2), a multifunctional mitochondrial aminotransferase, has diverse functions in cellular physiology and its products and substrates are biomarkers of renal, cardiovascular and metabolic diseases 26. Therefore, it was assumed these three hub genes played functional roles in suppressing cancer mainly through metabolic related pathways. Indeed, current study carried out GSEA using KEGG pathways database and found that majority of gene sets involved in metabolic related pathways. Consistently, TCGA Research Network illustrated that epigenetic reprogramming and oncogenic metabolism are the fundamental features of ccRCC. It is known that renal cancer is considered as one of the most deliberated and exemplary of malignancies characterized through metabolic reprogramming 27, 28. And genes mutated in renal cancer are complicated in a quantity of disparate pathways regulating various aspects of cellular metabolism, such as iron sensing and/or oxygen, the tricarboxylic acid (TCA) cycle, tumor energetics and glutamine metabolism 20, 29, 30. When translated to clinical scenarios, these three hub genes would have clinical values for diagnosis and personalized therapy for ccRCC.

Several limitations ought to be noted. In present study, some risk factors like gender, age, tumor grade, metastasis, and pathological stages were analyzed in patients suffered from ccRCC. However, other major established risk factors for ccRCC, such as hypertension and cigarette smoking, were not displayed for analysis during data collection. Additionally, more high quality ccRCC samples are needed to confirm our findings and elucidate the deep possible mechanisms of the effect on pathological stages.

In conclusion, our study identified and validated three novel hub genes including *EHHADH*, *ACADM* and *AGXT2*. Moreover, these three hub genes were found negatively correlated with clinical stages and having impact on patients' survival. Our novel data suggests these abnormally expressed or methylated genes could be used as therapeutic targets and biomarkers for ccRCC patients to be precisely diagnosed and effectively treated.

## Figures and Tables

**Table 1:** Hub genes in the module related with pathological stage.

Gene	Probe	Co-expression analysis (cor.geneModuleMembership)	Hub gene in PPI network	DEG analysis	
				logFC	p-value
EHHADH	205222_at	0.88	YES	-1.14	3.06E-04
ACADM	202502_at	0.89	YES	-1.10	8.19E-07
AGXT2	229229_at	0.86	YES	-2.37	5.67E-07
BBOX1	243018_at	0.93	NO	-2.24	1.35E-03
HAO2	220801_s_at	0.93	NO	-3.15	1.82E-10
ANK3	221751_at	0.83	NO	-1.26	1.18E-05
GOT1	1553878_at	0.87	NO	-1.18	1.24E-06
PHYH	203335_at	0.81	NO	-1.28	4.37E-10
SLC27A2	205768_s_at	0.82	NO	-2.49	2.19E-05
SERPINA5	209443_at	-0.81	NO	-4.00	2.12E-21
DDC	214347_s_at	0.83	NO	-2.45	5.30E-07
HIBCH	203711_s_at	0.88	NO	-1.47	6.50E-07
FBP1	205014_at	0.84	NO	-2.80	1.62E-11
HMGCS2	240110_at	0.80	NO	-1.87	1.54E-04
DMGDH	231591_at	0.84	NO	-1.51	5.66E-04
LRP2	205710_at	0.89	NO	-1.66	1.44E-03
CUBN	206775_at	0.80	NO	-1.85	9.03E-05
ECHS1	201135_at	0.81	NO	-1.45	1.34E-14
